# Postnatal development of collagen structure in ovine articular cartilage

**DOI:** 10.1186/1471-213X-10-62

**Published:** 2010-06-07

**Authors:** Mark C van Turnhout, Henk Schipper, Bas Engel, Willem Buist, Sander Kranenbarg, Johan L van Leeuwen

**Affiliations:** 1 Wageningen University, Department of Animal Sciences, Experimental Zoology Group, PO Box 338, 6700 AH, Wageningen, the Netherlands; 2 Wageningen University, Biometris, PO Box 100, 6700 AC Wageningen, the Netherlands; 3 Wageningen University & Research, Biometris, PO Box 100, 6700 AC Wageningen, the Netherlands

## Abstract

**Background:**

Articular cartilage (AC) is the layer of tissue that covers the articulating ends of the bones in diarthrodial joints. Across species, adult AC shows an arcade-like structure with collagen predominantly perpendicular to the subchondral bone near the bone, and collagen predominantly parallel to the articular surface near the articular surface. Recent studies into collagen fibre orientation in stillborn and juvenile animals showed that this structure is absent at birth. Since the collagen structure is an important factor for AC mechanics, the absence of the adult Benninghoff structure has implications for perinatal AC mechanobiology. The current objective is to quantify the dynamics of collagen network development in a model animal from birth to maturity. We further aim to show the presence or absence of zonal differentiation at birth, and to assess differences in collagen network development between different anatomical sites of a single joint surface. We use quantitative polarised light microscopy to investigate properties of the collagen network and we use the sheep (*Ovis aries*) as our model animal.

**Results:**

Predominant collagen orientation is parallel to the articular surface throughout the tissue depth for perinatal cartilage. This remodels to the Benninghoff structure before the sheep reach sexual maturity. Remodelling of predominant collagen orientation starts at a depth just below the future transitional zone. Tissue retardance shows a minimum near the articular surface at all ages, which indicates the presence of zonal differentiation at all ages. The absolute position of this minimum does change between birth and maturity. Between different anatomical sites, we find differences in the dynamics of collagen remodelling, but no differences in adult collagen structure.

**Conclusions:**

The collagen network in articular cartilage remodels between birth and sexual maturity from a network with predominant orientation parallel to the articular surface to a Benninghoff network. The retardance minimum near, but not at, the articular surface at all ages shows that a zonal differentiation is already present in the perinatal animals. In these animals, the zonal differentiation can not be correlated to the collagen network orientation. We find no difference in adult collagen structure in the nearly congruent metacarpophalangeal joint, but we do find differences in the dynamics of collagen network remodelling.

## Background

The articulating ends of bones in the diarthrodial joints are covered with a thin layer of articular cartilage (AC). During early development AC functions as a surface growth plate for the underlying bone [1-3]. In adult life, AC functions as a load bearing surface that transmits loads and provides a low friction environment. Between birth and skeletal maturity, AC has to accommodate both developmental and load bearing demands.

AC has been shown to develop from a fairly homogeneous tissue to a tissue with site specific composition [1,4,5] and site specific mechanical properties [6]. It is accepted that this development is affected by mechanical loads [7-9] that vary over the joint surfaces [10-12]. It is reasonable to assume that mechanical loads also affect the development of the collagen network, and that the differences in mechanical properties in adult life partly reflect differences in collagen network.

It is well known that the composition of the collagen network in AC changes during postnatal development: e.g. collagen type I is replaced by collagen type II [13-15], and the amount of collagen increases [5,16,17]. Structural remodelling of the collagen network in terms of the three zones has also been described for postnatal development [1,18]. However, the remodelling capacity of the collagen network in adult AC is limited [8,19-23]. Therefore, differences in collagen structure in adult AC are most likely already present at birth, or developed during postnatal cartilage maturation.

In adults, collagen fibre orientation and organisation are important parameters for the mechanical functions of AC [7,18,24,25]. The collagen network partly determines AC stiffness [26-29] and Poisson ratio [30] and in transient loads it also influences interstitial fluid flow [31-34]. Across species and anatomical sites, adult AC shows what is known as 'a Benninghoff structure' [1,17,18,25,35-37]. This structure is characterised by three layers in AC: from articular surface to tidemark there is first a thin layer with collagen fibres mainly oriented parallel to the articular surface, second there is a thicker transitional zone where the collagen fibres appear to lack a predominant orientation, and third there is the deep zone where collagen fibres are mainly oriented perpendicular to the tidemark.

Recently, (semi) quantitative studies into collagen fibre orientation in stillborn and juvenile animals versus adults have confirmed that the Benninghoff structure is absent at birth and that it is developed during early postnatal life [17,36,38]. Van Turnhout et al. [36] looked at equine AC in stillborn and mature animals. They found that collagen fibre orientation in the stillborn animals was predominantly parallel to the articular surface throughout the complete cartilage layer. In the adult animals, they confirmed the Benninghoff structure. They also noticed that some zonal differentiation was already present in the stillborn AC, although this was not reflected in the depth-dependent collagen orientation. Rieppo et al. [17] looked at juvenile and adult porcine AC. For the juvenile animals, they also found that a major part of the collagen fibrils was predominantly oriented parallel to the articular surface throughout the complete cartilage layer. In the adult animals, they confirmed the Benninghoff structure. Julkunen et al. [38] looked at rabbit AC from age 4 weeks to 18 months at six time points. Their results confirm the findings by Van Turnhout et al. [36] and Rieppo et al. [17].

Since the collagen network is an important factor for AC mechanics, these 'remarkable structural alterations during development and growth' [39] in part explain the stiffening of the AC layer during development [39]. Investigations into how the collagen network affects cartilage mechanics during postnatal development and the mechanical implications of the absence of a Benninghoff structure, can be performed with fibril reinforced finite element models, e.g. [40-42], provided that we have (quantitative) data on the collagen network at the time points of interest. Knowledge on the dynamics of collagen network development is also important for the validation of collagen remodelling algorithms, e.g. [43]. And finite element modelling of the time line of collagen structure development in AC will provide insight into the mechanical environment that goes with this development. Finally, better insight into the mechanobiology of cartilage development will help to mimic this process in vitro, which is important in the field of cartilage tissue engineering.

Last year's studies [17,36,38] showed evidence of postnatal collagen network remodelling in AC, but they had their limitations in showing its dynamics. Van Turnhout et al. [36] used two sample points (stillborn and adult) and had a limited number of animals. Rieppo et al. [17] used three sample points (4 months, 11 months and 21 months) that did not include perinatal animals. The study by Julkunen et al. [38] does contain more and earlier sample points in a time resolved manner. However, perinatal animals were not included in this study and the number of early sample points is still too limited to convincingly show the dynamics of collagen structure remodelling. These authors did show differences in collagen structure development between different joint surfaces (femur and tibia).

The objective of the present research is therefore to quantify the development of the collagen network in a model animal from birth to maturity. We further aim to shed more light on the zonal differentiation at birth as noted by Van Turnhout et al. [36], and to assess differences in (the development of) the collagen network between different anatomical sites of a single joint surface. We will look at anatomical sites that are expected to have different loading histories. We use quantitative polarised light microscopy (qPLM), sometimes called 'the gold standard of histology' [44,45], to investigate properties of the collagen network and we use the sheep (*Ovis aries*) as our model animal.

## Results

**Figure 1:**
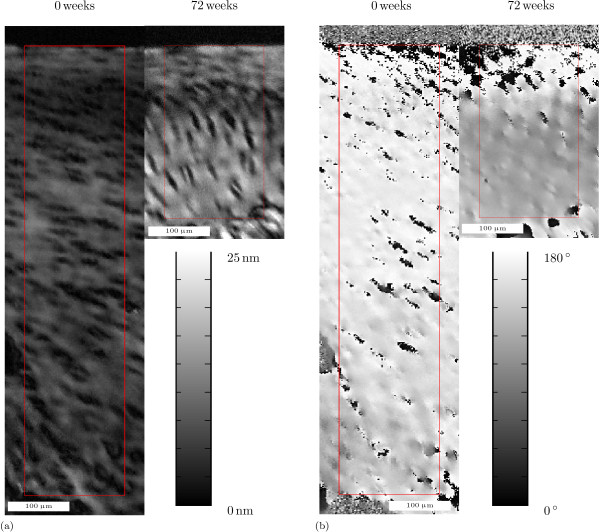
**Example of qPLM results**. Example of qPLM results with regions of interest at 0 weeks and 72 weeks of age. The width of each region of interest is 101 pixels = 161 μm. The articular surface is at the top of the figures. With (a) retardance, and (b) azimuth.

Figure 1 shows an example of qPLM results at 0 weeks and 72 weeks. As expected, cartilage thickness decreases, retardance increases (figure 1a) and predominant collagen orientation changes (figure 1b) between these ages. Examples of qPLM results at all ten ages are provided in additional file 1. The total number of samples was 1122. For each tested variable, the differences associated with the factor age (*A*_*j*_) are much larger than differences between the different anatomical sites. The following sections provide the average results per age and details on (significant) site-dependent differences.

### Cartilage thickness

The final model for the covariate cartilage thickness *D *with log-link and gamma variance function on the log scale is:(1)

**Figure 2:**
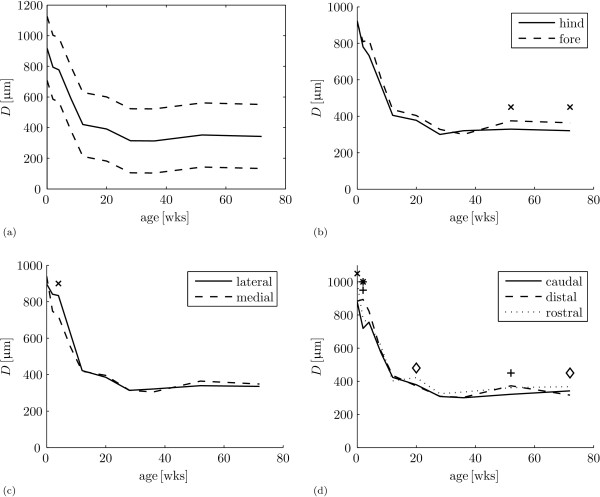
**Cartilage thickness**. (a) Mean cartilage thickness *D *(solid) ± standard deviation (dashed) for all samples at each age. (b) Mean cartilage thickness *D *as a function of age for hind legs (solid) and fore legs (dashed). The model predicts significant differences for: 'x' - fore leg > hind leg. (c) Mean cartilage thickness *D *as a function of age for lateral site (solid) and medial site (dashed). The model predicts significant differences for: 'x' - lateral site > medial site. (d) Mean cartilage thickness *D *as a function of age for the caudal site (solid), distal site (dashed) and rostral site (dotted). The model predicts significant differences for: 'x' - rostral site > caudal site, '+' - distal site > caudal site, '*' - distal site > rostral site, and ' ◇ - rostral site > distal site.

The results are gathered in table 1 and figure 2. As expected, there is a main effect of cartilage thickness *D *with age (table 1: *A*_*j*_, *p *< 0.001). *D *decreases in early life (figure 2a) such that at 28 weeks, *D *is at 34.6% ± 2.10% of its thickness at 0 weeks. No significant changes in *D *occur after 28 weeks (figure 2a): at 72 weeks *D *is at 37.6% ± 2.07% of its thickness at 0 weeks.

**Table 1:** Results for the statistical model for cartilage depth

fixed term	*A*_*j*_	*B*_*k*_	*C*_*l*_	*D*_*m*_	(*AB*)_*jk*_	(*AC*)_*jl*_	(*AD*)_*jm*_
***p*-value**	< 0.001	< 0.001	0.053	< 0.001	0.021	< 0.001	< 0.001

Results for the statistical model for cartilage depth *d *with log-link and gamma variance function. Letters for the fixed terms represent: *A*_*j *_- age, *B*_*k *_- fore/hind, *C*_*l *_- lateral/medial and *D*_*m *_- caudal/distal/rostral. Combinations represent interactions between these factors.

For the factor hind leg/fore leg, there is a main effect (table 1: *B*_*k*_, *p *< 0.001) and an interaction with age (table 1: (*AB*)_*jk*_, *p *= 0.021). For the main effect, the model predicts that cartilage is 6.40% ± 2.15% thicker in the fore legs than in the hind legs. For the interaction with age, cartilage in the fore legs is thicker than the cartilage in the hind legs at all ages but 0 weeks and 36 weeks (hind legs thicker, figure 2b). The interaction with age results in significant differences at 52 weeks (fore legs 14.3% ± 7.29% thicker) and 72 weeks (fore legs 13.7% ± 7.25% thicker).

For the factor lateral site/medial site, there is no main effect (table 1: *C*_*l*_, *p *= 0.053), but there is an interaction with age (table 1: (*AC*)_*jl*_, *p *< 0.001). This interaction does not show a trend (figure 2c) and is only significant at age 4 weeks (lateral site 15.8% ± 7.40% thicker).

For the factor caudal site/distal site/rostral site, there is a main effect (table 1: *D*_*m*_, *p *< 0.001) and an interaction with age (table 1: (*AD*)_*jm*_, *p *< 0.001). For the main effect, the model predicts that cartilage is 4.50% ± 2.12% thicker at the distal site than at the caudal site, and cartilage is 7.36% ± 2.18% thicker at the rostral site than at the caudal site. The interaction with age does not show a trend (figure 2d), but there are significant differences at ages 0 weeks (rostral 14.1% ± 7.31% thicker than caudal), 2 weeks (distal 25.0% ± 8.00% thicker than caudal, and distal 14.6% ± 7.33% thicker than rostral), 20 weeks (rostral 14.3% ± 7.32% thicker than distal), 52 weeks (distal 16.3% ± 7.45% thicker than caudal) and 72 weeks (rostral 16.3% ± 7.43% thicker than distal).

In the following sections, we present overviews of azimuth and retardance as a function of age. For these figures, cartilage depth as a function of age is estimated from an exponential fit through the data for all samples. This fit is given by(2)

with *D*_*f *_the cartilage depth in μm and *t *the age in weeks.

### Collagen orientation

**Figure 3:**
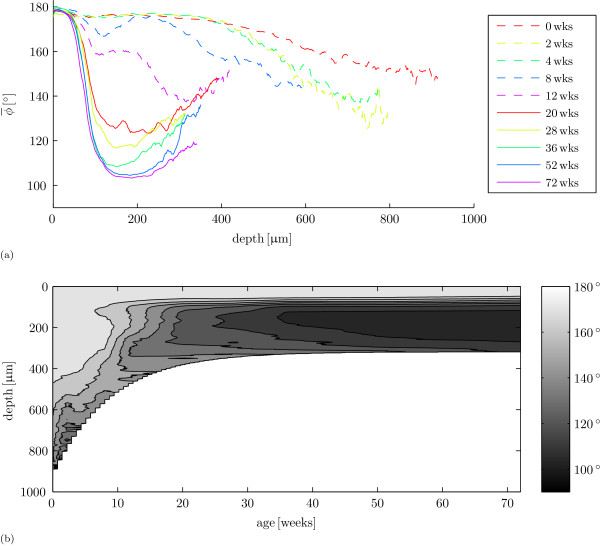
**Predominant collagen orientation per age**. (a) Mean predominant collagen orientation  as a function of age and cartilage depth. Colours represent age in weeks. (b) Overview of the results per age in a contour plot.

Predominant collagen orientation changes with age (figure 3). Predominant collagen orientation is parallel to the articular surface for most of the cartilage depth for the youngest animals. As age increases, predominant collagen orientation in the deep cartilage changes, first at a depth where the future transitional zone will appear for animals of age 8 weeks and 12 weeks. At the age of 20 weeks, we first see a Benninghoff structure: predominant collagen orientation changes from parallel to the articular surface to a predominant orientation more towards the perpendicular with the subchondral bone in the deep cartilage. With increasing age, predominant collagen orientation in the deep cartilage aligns further towards the perpendicular to the cartilage/bone interface. The patterns in figure 3a appear to converge to an azimuth between 120° and 160° near the cartilage/bone interface. Thus for the youngest samples, azimuth decreases in the deep zone near the cartilage/bone interface, and in the older samples, azimuth increases in the deep zone near the cartilage/bone interface. Figure 3b shows an overview of the dynamics of the azimuth results for all samples (sites) per age taken together (interpolated from figure 3a, with depth *D*_*f*_). The final model for the covariate orientation index  with logit-link and binomial variance function on the logit scale is:(3)

**Figure 4:**
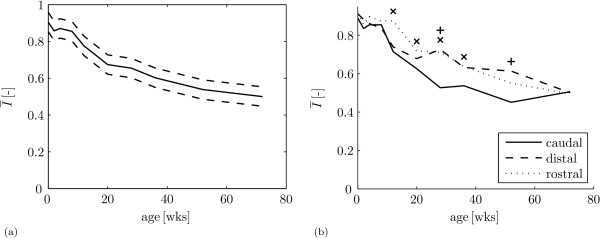
**Orientation index**. (a) Mean orientation index  (solid) ± standard deviation (dashed) as a function of age. (b) Mean orientation index  as a function of age for the caudal site (solid), distal site (dashed) and rostral site (dotted). The model predicts significant differences for: 'x' - rostral site > caudal site and '+' - distal site > caudal site.

The results are gathered in table 2 and figure 4. There is a main affect of age on the orientation index  (table 2: *A*_*j*_, *p *< 0.001). As expected,  is close to 1 for the youngest animals and decreases with age (figure 4a), as less collagen fibres are predominantly oriented parallel to the articular surface. At 20 weeks,  is 0.21 lower than at 0 weeks. After 20 weeks, no significant differences between successive age groups occur, although  at 72 weeks (0.41 lower than at 0 weeks) does differ significantly from  at 36 weeks (0.31 lower than at 0 weeks).

**Table 2:** Results for the statistical model for orientation index

fixed term	*A*_*j*_	*B*_*k*_	*C*_*l*_	*D*_*m*_	(*AD*)_*jm*_
***p*-value**	< 0.001	0.560	0.013	< 0.001	0.011

Results for the statistical model for orientation index  with logit-link and binomial variance function. Letters for the fixed terms represent: *A*_*j *_- age, *B*_*k *_- fore/hind, *C*_*l *_- lateral/medial and *D*_*m *_- caudal/distal/rostral. Combinations represent interactions between these factors.

There is a main effect for lateral sites versus medial sites (table 2: *C*_*l*_, *p *= 0.013). The model shows that  is 0.22 higher at the lateral site. Inspection of the average orientation patterns for these sites per age (additional file 2) indicates two differences: 1) the medial site appears to be further in the development of the Benninghoff structure at ages 4 weeks, 12 weeks and 52 weeks; and 2) the depth where the predominant collagen fibre orientation is halfway in the transition from superficial zone to deep zone, is smaller for the medial site than for the lateral site at ages 28 weeks, 36 weeks and 52 weeks. Both effects result in a lower value of .

For the factor caudal site/distal site/rostral site, there is a main effect (table 2: *D*_*m*_, *p *< 0.001) and an interaction with age (table 2: (*AD*)_*jm*_, *p *= 0.011). For the main effect, the model predicts that  is 0.06 higher at the distal site than at the caudal site, and  is 0.08 higher at the rostral site than at the caudal site. The interaction with age shows significant differences between 12 weeks and 52 weeks (figure 4b).  is higher at the rostral site than at the caudal site at 12 weeks (0.16), 20 weeks (0.09), 28 weeks (0.20) and 36 weeks (0.10).  is higher at the distal site than at the caudal site at 28 weeks (0.21) and 52 weeks (0.17). Inspection of the average orientation patterns for these sites per age (additional file 3) shows that the caudal site develops its Benninghoff structure earlier in life than the rostral and distal site: predominant collagen orientation at a depth of 100 μm at 28 weeks is 104° at the caudal site against 127° at the rostral site and 125° at the distal site. The average orientation patterns do not show a trend for differences between development for the rostral site and distal site. At 72 weeks of age, all site-dependent variation in collagen orientation has vanished (figure 4b).

### Retardance

**Figure 5:**
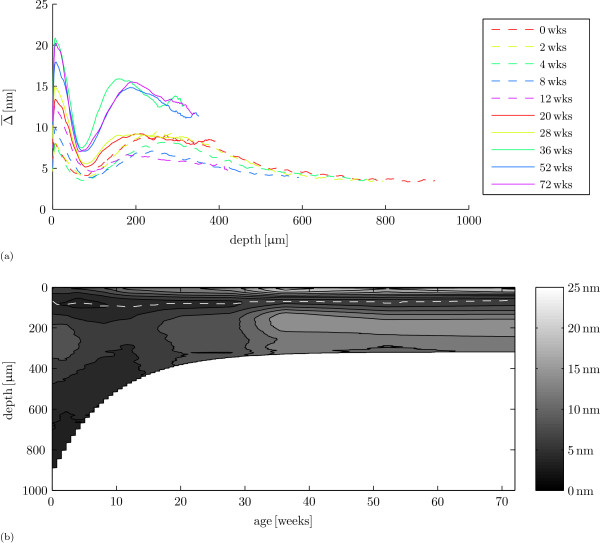
**Retardance per age**. (a) Mean sample retardance as a function of cartilage depth. Colours represent age in weeks. (b) overview of the results in a contour plot. The dashed white line marks the retardance minimum.

The retardance values change during development, but the general retardance patterns appear indifferent to age (figure 5). For all ages from articular surface towards the bone, retardance first has a maximum near the articular surface, followed by a minimum at a depth of ≈ 50 μm that is in turn followed by another maximum deeper in the cartilage (figure 5a). Figure 5b shows an overview of the dynamics of the retardance results for all samples (sites) per age taken together (interpolated from figure 5a, with depth *D*_*f*_). The position of the retardance minimum is marked with a dashed white line.

The final model for the maximum retardance near the articular surface Δ_max _with log-link and gamma variance function on the log scale is:(4)

**Figure 6:**
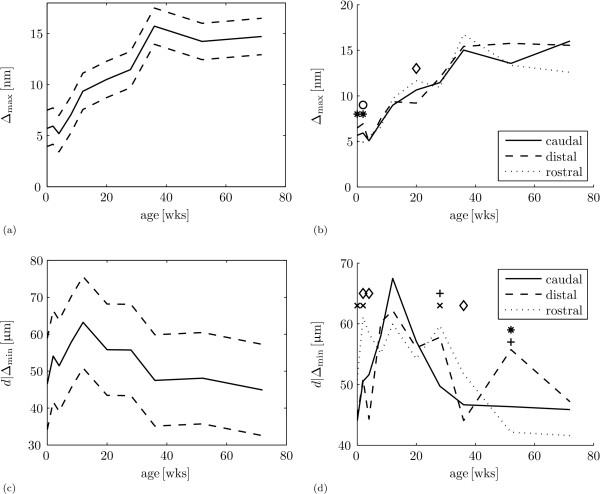
**Retardance maximum and minimum**. (a) Mean of the maximum retardance near the articular surface Δ_max _(solid) ± standard deviation (dashed) as a function of age. (b) Mean of the maximum retardance near the articular surface Δ_max _as a function of age for the caudal site (solid), distal site (dashed) and rostral site (dotted). The model predicts significant differences for: '*' - distal site > rostral site, '◇' - rostral site > distal site, and 'o' - caudal site > rostral site. (c) Mean position of the retardance minimum near the articular surface *d*|Δ_min _(solid) ± standard deviation (dashed) as a function of age. (d) Mean position of the retardance minimum near the articular surface *d*|Δ_min _as a function of age for the caudal site (solid), distal site (dashed) and rostral site (dotted). The model predicts significant differences for: 'x' - rostral site > caudal site, '+' - distal site > caudal site, '*' - distal site > rostral site, and '◇' - rostral site > distal site.

The results are gathered in table 3 and figure 6. There is a main effect of age with the maximum retardance near the articular surface Δ_max _(table 3: *A*_*j*_, *p *< 0.001). Δ_max _increases with age until 36 weeks when it is 164% ± 20.0% higher than at 0 weeks (figure 6a). No significant changes occur after 36 weeks: at 72 weeks, Δ_max _is 154% ± 19.3% higher than at 0 weeks,

**Table 3:** Results for the statistical model for the maximum retardance

fixed term	A_*j*_	B_*k*_	C_*l*_	D_*m*_	(*AD*)_*jm*_	(*BD*)_*km*_
*p*-value	< 0.001	0.172	0.396	0.179	0.034	0.005

Results for the statistical model for the maximum retardance near the articular surface Δ_max _with log-link and gamma variance function. Letters for the fixed terms represent: *A*_*j *_- age, *B*_*k *_- fore/hind, *C*_*l *_- lateral/medial and *D*_*m *_- caudal/distal/rostral. Combinations represent interactions between these factors.

There are no other main effects, but there are two significant interactions with the factor caudal site/distal site/rostral site (table 3: (*AD*)_*jm*_, *p *= 0.034 and (*BD*)_*km*_, *p *= 0.005). The interaction with age (*AD*)_*jm *_shows significant differences at ages 0 weeks (distal 30.3% ± 12.8% higher than rostral), 2 weeks (caudal 25.0% ± 12.2% higher than rostral, and distal 41.3% ± 13.8% higher than rostral) and 20 weeks (rostral 28.4% ± 12.6% higher than distal), but no trend (figure 6b). The interaction of caudal site/distal site/rostral site with hind legs/fore legs is due to the caudal site: Δ_max _is 18.7% ± 4.92%, higher in the hind legs than in the fore legs for the caudal site. The model shows no differences between hind legs and fore legs for the rostral and distal site.

The final model for the position of the retardance minimum near the articular surface *d*|Δ_min _with log-link and gamma variance function on the log scale is the same as for the orientation index (equation 3). There is a main effect of the factor age on *d*|Δ_min _(table 4: *A*_*j*_, *p *= 0.003). With increasing age, *d*| Δ_min _first increases until 12 weeks and next deceases until 72 weeks of age (figure 6c). At the maximum at 12 weeks, *d*| Δ_min _is 35.4% ± 7.63% higher than at 0 weeks. At 36 weeks of age an older, the model shows no difference for *d*|Δ_min _with 0 weeks of age.

**Table 4:** Results for the statistical model for the position of the retardance minimum near the articular surface

fixed term	A_*j*_	B_*k*_	C_*l*_	D_*m*_	(*AD*)_*jm*_
*p*-value	0.003	0.733	0.002	0.536	< 0.001

Results for the statistical model for the position of the retardance minimum near the articular surface *d*|Δ_min _with log-link and gamma variance function. Letters for the fixed terms represent: *A*_*j *_- age, *B*_*k *_- fore/hind, *C*_*l *_- lateral/medial and *D*_*m *_- caudal/distal/rostral. Combinations represent interactions between these factors.

The interaction of caudal site/distal site/rostral site with age (table 4: (*AD*)_*jm*_, *p *< 0.001) has significant differences at ages 0 weeks (rostral 17.2% ± 8.59% higher than caudal), 2 weeks (rostral 19.1% ± 8.72% higher than caudal, and rostral 21.7% ± 8.91% higher than distal), 4 weeks (rostral 28.4% ± 9.40% higher than distal), 28 weeks (rostral 17.4% ± 8.59% higher than caudal, and distal 16.4% ± 8.53% higher than caudal), 36 weeks (rostral 17.7% ± 8.62% higher than distal) and 52 weeks (distal 18.7% ± 8.69% higher than caudal, and distal 30.1% ± 9.52% higher than rostral), but these effect do not show a trend (figure 6d). There is also a significant main effect for lateral site/medial site (table 4: *C*_*l*_, *p *= 0.002): *d*|Δ_min _is 7.14% ± 2.31% higher at the lateral site.

## Discussion

The results on cartilage thickness are mostly in line with previous work. Brommer et al. [6] for instance, analysed site-dependent development of cartilage thickness in the equine proximal phalanx. As we did in our study, they found that cartilage thickness decreases during development, and they found no site-dependent differences in cartilage thickness in mature animals. In our study, cartilage at the caudal site is thinner than at the rostral and distal site and cartilage in the fore legs is thicker than in the hind legs, although the differences are small (< 8%). Cartilage thickness has been shown to respond to mechanical loads during development [7,46-48] and the results for the development of the collagen network suggest influences of different loading regimes at different anatomical sites. However, cartilage thickness in the mature animal is thought to relate to joint congruency, i.e. the similarity of the two articulating cartilage surfaces [49,50]. This explains why we find only small site-dependent differences in cartilage thickness in the nearly congruent joint that we investigated. This leaves us with the difference in cartilage thickness between hind legs and fore legs. Contrary to the remark by Simon [51] that cartilage is in general thicker in the hind limbs, we find that cartilage is thicker in the fore limbs, and that the difference increases slightly between birth and maturity.

As in previous studies on collagen structure in horses [36], pigs [17] and rabbits [38], we find that the perinatal predominant collagen fibre orientation is parallel to the articular surface throughout most of the cartilage depth and that this arrangement changes to an arcade-like Benninghoff structure as the cartilage matures. We also confirmed the presence of a retardance minimum in the perinatal animals as noted earlier by Van Turnhout et al. [36]. The collagen remodelling process appears finished at 36 weeks of age in our sheep in this joint (e.g. figures 3b and 5b): the differences in qPLM results are small after this age and three of the four measured parameters (*D*, Δ_max_, and *d*|Δ_min_) do not change significantly between 36 weeks and 72 weeks of age. Hunziker et al. [1] found no noteworthy changes after puberty in the structural organization of rabbit AC and 36 weeks is also approximately when the sheep are sexually mature: the first ovulation or puberty in the sheep occurs at ≈ 6 months of age or at a mass of 30 kg to 35 kg, and this transition lasts ≈ 5 weeks [52]. It is well before skeletal maturity, which occurs around 12 months of age [53-55]. Also, the slaughter mass results (additional file 4) show that the animals still developed after the age of 36 weeks.

The fact that collagen orientation appears to vary in the deep zone (figure 3a) is related to our decision to average sample profiles up to the mean depth of the sample pool. There are samples that are shorter than this mean depth and the number of samples that we (can) use to average therefore decreases from the minimum depth in the sample pool to the mean depth. The benefit of this procedure is that it better captures the superficial and transitional zones: e.g., the observation that the absolute depth of the retardance minimum appears invariant to age is evident when analysed in this manner. The downside is that from the minimum depth to the mean depth in the sample pool, we have an increasing proportion of data that is very close to the cartilage/bone interface. The azimuth data in particular may deviate from the deep zone data at the cartilage/bone interface, where the collagen ensures the integration of these two tissues. Inspection of the individual orientation patterns has shown that the azimuth is in fact fairly constant in the deep zone (additional file 5), and that the deviations towards the cartilage/bone interface in figure 3a are largely due to the increasing influence of cartilage/bone interfaces as described above. We find site dependent differences in collagen network remodelling (additional files 2 and 3). Both  and *d*|Δ_min _are higher at the lateral site than at the medial site (tables 2 and 4, *C*_*l*_, *p *= 0.013 and *p *= 0.002).  is higher for less advanced Benninghoff structures and for Benninghoff structures with higher *d*|Δ_min_. The results for *d*|Δ_min _suggest that its smaller value for the medial sites is correlated to the smaller values of . Thus, the transitional zone is at a larger depth at the lateral side, i.e. the superficial zone is thicker at the lateral side.

Also, the caudal site develops different from the distal and rostral sites: it develops its Benninghoff structure earlier in life (figure 4b, additional file 2) and maximum retardance is higher in the hind legs for the caudal site, but not for the distal and rostral site (table 3, (*BD*)_*km*_, *p *= 0.005). These observations suggests a loading regime during development that is different for the caudal sites compared to the rostral and distal sites. In the last sample point however, we find no site dependent differences in predominant collagen orientation patterns (additional file 2) or the orientation index  (figure 4b). Gründer [56] reports a faster structural development for sheep tibial AC compared to femural AC, Julkunen et al. [38] showed that collagen structure development and adult collagen structure both differ between femur and tibia in rabbits, and Brama et al. [57] showed changes in collagen structure in juvenile foals as a result of exercise training. However, Egger et al. [25] did not find differences in collagen structure in adult elephant knee cartilage and they propose that this is due to the congruency in the elephant knee. This hypothesis is supported by our data: in the examined nearly congruent joint that was subjected to physiological loads, we also find no differences in collagen orientation between sites on the same cartilage surface for the oldest (adult) animals.

Our data shows that the predominant collagen orientation first changes at a depth that marks the proximal boundary of the transitional zone in the mature animal (figure 3a). The resulting intermediate pattern for predominant collagen orientation that we find at 12 weeks, is similar to the intermediate pattern found in a 4.5 month year old horse [36], in 11 month old pigs [17], and in rabbits of 4 weeks to 6 weeks [38]. This intermediate pattern is present for all anatomical sites in the current study. This suggests that the depth-dependent pattern of collagen remodelling is similar across species, joints and anatomical sites, except for the timing (e.g. earlier development for the caudal site in this study, figure 4b and additional file 2). The difference in species (porcine versus ovine) and anatomical location (metacarpus versus knee) can explain why Rieppo et al. [17] found that collagen remodelling was not finished when their pigs reached sexual maturity.

The absence of a Benninghoff collagen structure at birth may have implications for postnatal morphogenesis. Results of simulations with finite element models [58,59] and *in vitro *experimental results [60] show correlations between hydrostatic pressure and inhibition of ossification and between shear strains and promotion of ossification. An effect of the Benninghoff collagen structure is that it limits cartilage swelling, and it thereby effectively increases the hydrostatic (osmotic) pressure in the deep zone [61-63]. The development of the Benninghoff structure might thus serve to prevent ossification and maintain a functional cartilage layer in the adult animal. It would be interesting to see if cartilage ossification is promoted when the Benninghoff collagen structure cannot develop.

The retardance minimum near the articular surface is traditionally associated with the transitional zone, a layer with a weak anisotropic collagen arrangement, in adult animals [18,35]. Our special interest in this retardance minimum appears to have been justified. Not only is it present at birth without evidence of differentiation in collagen orientation, which is confirmed by earlier work on equine [36] and porcine cartilage [17], it is also the only parameter that is similar in the youngest and oldest animals (figure 6c). During cartilage maturation, its thickness decreases to about one third of the perinatal thickness; collagen fibre orientation changes from a network predominantly parallel to the articular surface over the cartilage depth to a Benninghoff structure; and maximum retardance increases to about twice that of neonatal cartilage. Compared to these developmental changes, the depth of the retardance minimum (≈ 50 μm) appears relatively indifferent to the remodelling process (figure 6c). Thus, a layer with a weak anisotropic collagen arrangement, or less collagen, or both, is already formed before birth. This can for instance be a result of *in utero *movement and muscular loads on the joint [64,65]. If so, then apparently the mechanical state in this layer does not change enough after birth to result in a much stronger anisotropic collagen fibre arrangement. If *in utero *joint loading is responsible for the emergence of this layer, we would predict its absence in animal models that e.g. lack skeletal muscle activity [66]. Finite element models may help to explain what is so special about this layer, and its location.

With this paper, we provide essential information for analysis of the role of the collagen fibre network during development, e.g. by fibril reinforced finite element models [40-42]. It is the first time that the dynamics of post natal remodelling of collagen fibre organisation have been time and space resolved from birth to maturity. Therefore, we were able to present the first solid observation of a retardance minimum in perinatal animals, more or less at a fixed distance (≈ 50 μm) from the articular surface. This is an important step towards a better understanding of the mechanobiology of articular cartilage development. With additional information on collagen densities during development, we will be able to interpret the retardance minimum as a region with less collagen or a region with more anisotropic collagen and see how much this interpretation changes during development. With additional information on the development of glycosaminoglycan concentrations and fixed charge densities in the AC, it becomes possible to estimate the mechanical environment that drives the depth-dependent AC development in general, and depth-dependent collagen orientation remodelling in particular.

## Conclusions

The collagen network in articular cartilage remodels between birth and sexual maturity from a network with predominant orientation parallel to the articular surface to a Benninghoff network. Changes in predominant collagen orientation occur first at a depth that marks the proximal boundary of the transitional zone in adult animals. This is the first solid observation of the dynamics of collagen structure remodelling, and provides important information for the further investigation of articular cartilage mechanobiology.

The fact that the retardance minimum near the articular surface is present at all ages shows that a zonal differentiation is already present in the perinatal animals. However, the zonal differentiation can not be correlated to changes in collagen network orientation in these animals. Furthermore, we were able to show that the depth of this retardance minimum is fairly constant during development. Without information on the development of collagen densities, we cannot tell if this retardance minimum is the result of a region with less collagen or a region with collagen with less anisotropic predominant orientations; and we cannot tell whether the relative contributions (less collagen and less anisotropic arrangement) change during development. We intent to address this in a future publication.

We observe differences in the dynamics of collagen remodelling between different anatomical sites, but in the adult animals we see no difference in collagen structure (i.e. they are all similar Benninghoff structures) or cartilage thickness. We expect that this is due to the congruency of the joint that we investigated. Comparison of the data in the current paper with other data on collagen remodelling in literature, suggests that the general pattern of collagen remodelling (i.e. where the first changes in collagen orientation occur) is similar between joints and species.

## Methods

### Animals

Animals were obtained from a local sheep farm. We obtained 50 female sheep for 9 sample points (five sheep for each sample point, and five spare sheep), at ages 2 weeks, 4 weeks, 8 weeks, 12 weeks, 20 weeks, 28 weeks, 36 weeks, 52 weeks and 72 weeks. An additional four stillborn lambs were used (labelled age = 0 weeks). Animals were kept at the farm with their mother until sacrifice or the age of 12 weeks. Animals that were 12 weeks old were collected at the farm and housed at the universities laboratory animal facility 'Ossekampen' until sacrifice.

Animals were kept together at pasture with ample space. They had ad lib access to water and food. The animals were born between 24th of February 2007 and 21st of March 2007. In September and October the disease blue tongue spread over the Netherlands and we had to house the animals temporarily inside a barn. Due to the disease, three infected animals had to be excluded from the experiment. All animals were closely monitored, and the other animals showed no clinical signs of blue tongue infection. A further three animals were excluded from the experiment due to accidents with a dog and a male sheep. Therefore, the total number of animals at the end of the experiment was 48, and the number of animals for the first sample point (0 weeks, stillborn) and the last sample point (72 weeks) was four. Animals were weighed prior to sacrifice. The experiment was approved was by the the Wageningen University Animal Experiments Committee.

### Sample preparation

Animals for the sample points from 2 weeks to 12 weeks were collected at the farm and sacrificed with an overdose of T61. Animals for the remaining sample points were brought to an abattoir to be sacrificed. The animals' legs were collected immediately following sacrifice. For each animal we randomly selected either the two left legs or the two right legs for qPLM analysis. At each sample point, the ratio left:right was either 3:2, 2:3 or 2:2.

**Figure 7:**
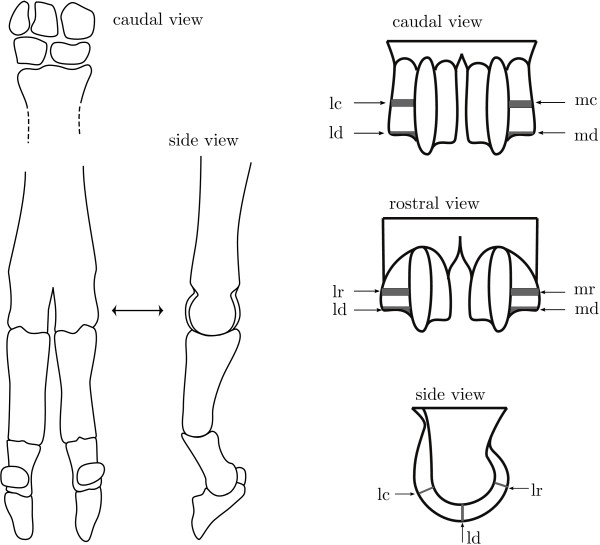
**Sketch of sample sites**. Left: sketch of the bones in the lower fore leg of a sheep. The double arrow shows the distal metacarpus that was used in this study. Right: sketch of anatomical sampling sites with l - lateral, m - medial, c - caudal, d - distal, and r - rostral.

Skin and subcutaneous tissue were removed from the metacarpophalangeal joints (figure 7) and they were carefully opened. Split line patterns were created on the articular surface of the distal metacarpus with a sharp round needle charged with Indian ink for two of the five animals per sample point (additional file 6). The needle was inserted perpendicular to the articular surface at 2 mm intervals and excess ink was removed by rinsing. The resulting split line patterns were recorded with a Nikon D-100 digital camera with a Micro-Nikon 55 mm objective and used to determine the cutting orientation for this sample point.

We used a dental saw to take the medial and lateral hemispheres from the distal end of each cannon bone. These hemispheres were fixed with formalin and decalcified with EDTA (10% EDTA, pH 7.4) until the hemispheres could be cut with a razor blade. The hemispheres were then divided into a rostral, a distal and a caudal sample (figure 7). Of these, the distal site is expected to be subject to a more static load and the rostral and caudal sites are expected to be subject to a more intermittent load during locomotion [6]. These samples were washed and infiltrated with sucrose (25% sucrose on PBS) overnight, snap frozen in liquid nitrogen and stored at -80°C until further processing, and finally cut parallel to the superficial split lines (as observed in two of the five animals) to 7 μm thick histological slices with a cryostat (Reichert 2800N).

### Quantitative polarised light microscopy

The birefringent material in cartilage are the fibrils in the collagen network and qPLM measures two parameters of birefringent structures: azimuth and retardance. The azimuth *φ *does not measure individual fibril orientations, but is the predominant orientation of the collagen fibres in the network in the pixel. The retardance Δ is a measure of the amount of birefringent material (e.g. collagen fibrils) that is associated with azimuth *φ *[67]: low retardance indicates one of three things: that 1) there is fewer collagen in this pixel; or that 2) the measured orientation belongs to a collagen network with a low level of anisotropy; or 3) a combination of these two.

Macroscopically normal histological samples were mounted with water and analysed with the LC-PolScope system for qPLM [68,69]. Images were obtained with a Zeiss Axiovert 200M microscope at a 5×/1.6 magnification, equipped with a Q-imaging monochrome HR Retiga EX 1350 camera. Recorded intensity images had a resolution of 1.59 μm^2^/pixel and were stored in 8 bit TIFF format. We used the five frame setting with background correction as described by [70]. The recorded images were analysed for predominant collagen fibril orientation and tissue retardance with custom written scripts implemented in Matlab (version 7.8.0 R2009a, The MathWorks, Inc., 1984-2005).

### Data analysis

#### Azimuth and Retardance patterns

**Figure 8:**
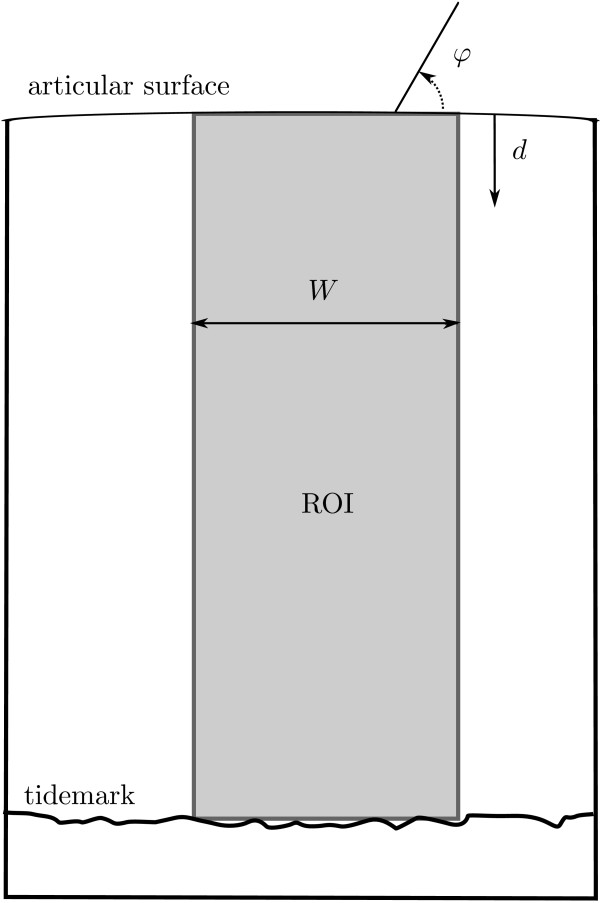
**Example for data analysis**. For each sample a region of interest (ROI) is extracted from the images. The width *W *of this region is 101 pixels, and it runs from *d *= 1 pixel at the articular surface to *d *= *D *pixels at the cartilage/bone interface. The predominant orientation *φ *is expressed relative to the articular surface. For sample pattern calculations, we used the 101 pixels over the width of the ROI at each depth *d *= 1, 2, 3, ..., D.

We extracted a rectangular region of interest (ROI) with a width of 101 pixels from the qPLM images. This ROI reaches from articular surface to the cartilage/bone interface, i.e. up to the calcified tissue in young animals, and up to the tidemark in older animals. The azimuth was expressed with respect to the articular surface (figure 8).

Retardance patterns as a function of height were obtained for each sample by taking the arithmetic mean over the width of 101 pixels at each depth *d*. These patterns were used to determine the maximum retardance near the articular surface and the position of the retardance minimum in the transitional zone. To obtain an average azimuth at depth *d *from 101 values with predominant fibril azimuth, the arithmetic mean did not suffice [71]. This is because the predominant orientation of two fibres with azimuth 1° and 179° is 0° (or 180°), and not 90°. We therefore introduced a retardance weighted average azimuth () that is at each depth *d *obtained by maximising the function *I*(5)

for *W *= 101 pixels over the width of the ROI, and for  on the interval 0 < ≤ π [36]. The summation over the absolute values of the inner dot product found the line with the smallest difference in angles compared to all *W *lines described by the azimuth values *φ*(*w*). The multiplication with the retardance Δ (*w*) assigned a smaller importance to the azimuth in pixels with low retardance, i.e. to pixels with less collagen associated with the predominant orientation *φ*(*w*) [36].

For statistical analysis of the orientation patterns we calculated for each sample an orientation index . This index is defined as the average dot product of the sample azimuth pattern with a reference pattern with continuous azimuth θ = 180°:(6)

with *D *the number of points (pixels) over the depth of the sample (figure 8).

#### Average azimuth patterns between samples

We used the sample patterns  to calculate average azimuth patterns  between samples. Because of differences in cartilage thickness, the number of samples that we can analyse decreases once we are at a depth larger than *D *for the shortest dataset in the sample pool. Thus, we maximised equation (7) to analyse the azimuth patterns at each depth *d *over the available samples *S*(*d*) at that depth *d*:(7)

with *s *the sample number,  on the interval 0 < ≤ π and *d *up to the mean cartilage thickness of the samples: 0 <*d *≤ .

### Statistical analysis

Data were analysed with generalized linear mixed models because some of the variables analysed are not normally distributed. Also, measurements on the same animal and position within an animal are dependent. This excludes conventional analyses such analysis of variance or regression that are intended for normally distributed and independent data. We therefore used the penalized quasi-likelihood methodology described by Schall [72], Breslow & Clayton [73] and Engel & Keen [74]. Calculations were performed with GenStat [75]. The models comprised random effects with associated components of variance, that allowed for dependence between observations of the same animals and the same anatomical sites. Thus, we used a nested structure within animal for hind leg/fore leg, lateral/medial and caudal/distal/rostral sites. In particular, this allowed for additional dependence within animals between duplicate observations on the same site. We are interested in the development of differences between the different anatomical sites with age. Therefore, fixed effects (systematic effects) comprised main effects and all second order interactions for factors age, hind leg/fore leg, lateral/medial site and caudal/distal/rostral site in the initial models. Models were fitted separately to four response variables: cartilage thickness *D*, the orientation index , the maximum retardance value near the articular surface Δ_max_, and the position of the retardance minimum near the articular surface *d*|Δ_min_. For the variables *D*, Δ_max _and *d*|Δ_min_, we used a log link and gamma variance function, with a multiplicative dispersion parameter. For the variable , we used a logit link (logit *p *= log *p/*(1 - *p*)) and binomial variance function with an additional multiplicative dispersion parameter. Random effects on the link scale were assumed to follow normal distributions. Tests were based on an approximate *F*-test [76] applied to the adjusted dependent variate from the last iteration step of the iterative re-weighted restricted maximum likelihood algorithm [74] that we used. The link functions provide the relationship between the linear predictor and the mean of the distribution function and the chosen link and variance functions were needed to achieve satisfactory (normally distributed) residuals for the models. Non-significant (*p *> 0.05) higher order interactions were dropped from the initial models. We used the following symbols in the models: *μ*: intercept; *A*_*j*_, *j *= 0, 2, 4, 8, 12, 20, 28, 36, 52, 72: age in weeks; *B*_*k*_, *k *= 1, 2: fixed factor hind leg/fore leg; *C*_*l*_, l = 1, 2: fixed factor lateral site/medial site; *D*_*m*_, *m *= 1, 2 3: fixed factor caudal site/distal site/rostral site; *L*_*i*_: random factor individual lamb; and (*LB*)_*ik*_, (*LBC*)_*ikl *_and (*LBCD*)_*iklm *_nested random factors within lamb. The final model that we fitted for each covariate, is presented in the results section. In the text, we quantify significant differences as mean ± standard error as predicted by the model. For the model with the logit link (for ), we can only present the mean, and not the standard errors. In the figures, we use raw means and associated standard deviations, and not model predictions, to illustrate the results.

## Authors' contributions

MvT carried out the design of the study and its coordination, the data processing, analysis and interpretation, and drafting the manuscript; and participated in acquisition of data and the statistical analysis. HS carried out the acquisition of data (histology and microscopy), and participated in data analysis and interpretation and drafting the manuscript. BE and WB carried out the statistical analysis and participated in the interpretation of data and drafting the manuscript. SK and JvL participated in the design of the study and its coordination, data analysis and interpretation and critical revisions of the manuscript. All authors read and approved the final manuscript.

## Supplementary Material

Additional file 1 **Examples of PLM images for all ten age points**. Top: azimuth results. Bottom: retardance results. Click here for file

Additional file 2 **Orientation patterns for caudal/distal/rostral sites**. Average orientation patterns for all ten ages divided in caudal, distal and rostral samples. Click here for file

Additional file 3 **Orientation patterns for lateral/medial sites**. Average orientation patterns for all ten ages divided in lateral and medial samples. Click here for file

Additional file 4 **Slaughter mass**. Slaughter mass together with the exponential fit. The mass at 36 weeks is an estimate from the butcher, because these animals were not weighed prior to sacrifice. Click here for file

Additional file 5 **Constant azimuth in the deep zone**. Illustration of the constant azimuth in the deep zone for a 72 week old animal. Left: azimuth PLM image with ROI. The articular surface is on top of the image. Right: corresponding orientation pattern. The azimuth is fairly constant in the deep zone up until the last 3% of the total depth. Click here for file

Additional file 6 **Illustration of split lines**. Left: lateral side of a left hind leg in a 2 week old lamb. Right: medial side of a left hind leg in a 72 week old lamb. Click here for file
