# Supplementary figures and images for: Postnatal development of collagen structure in ovine articular cartilage
Source: BMC Dev Biol. 2010 Jun 7;10:62. doi: 10.1186/1471-213X-10-62 (PMC2906441; doi:10.1186/1471-213X-10-62)

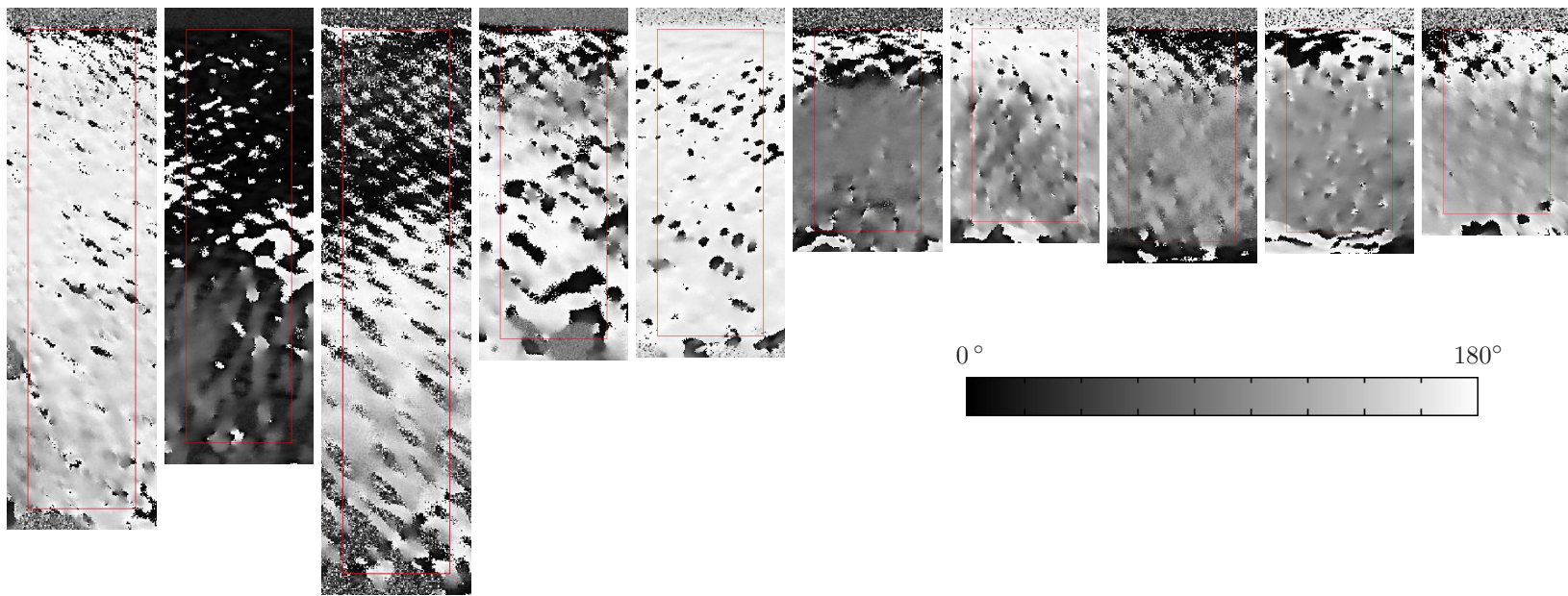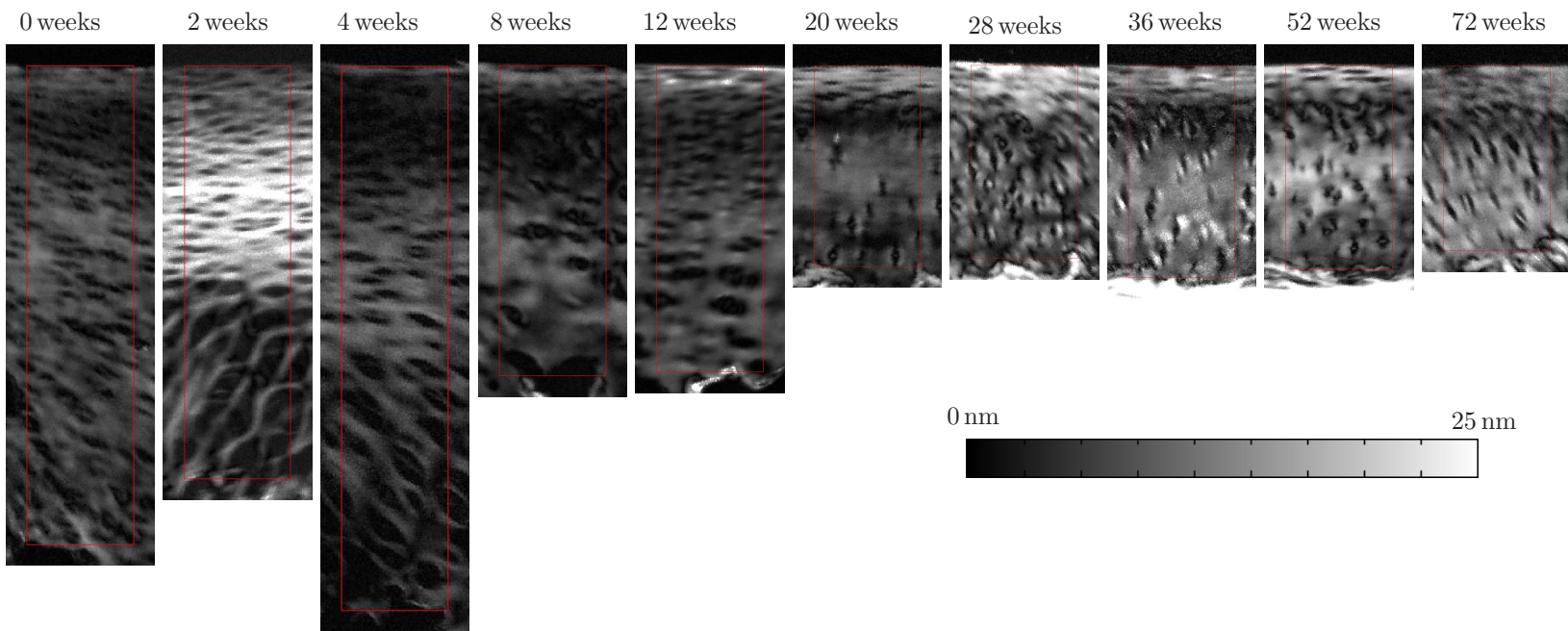

Supplement: Additional file 1 — Examples of PLM images for all ten age points. Top: azimuth results. Bottom: retardance results. [file 1471-213X-10-62-S1.PDF]

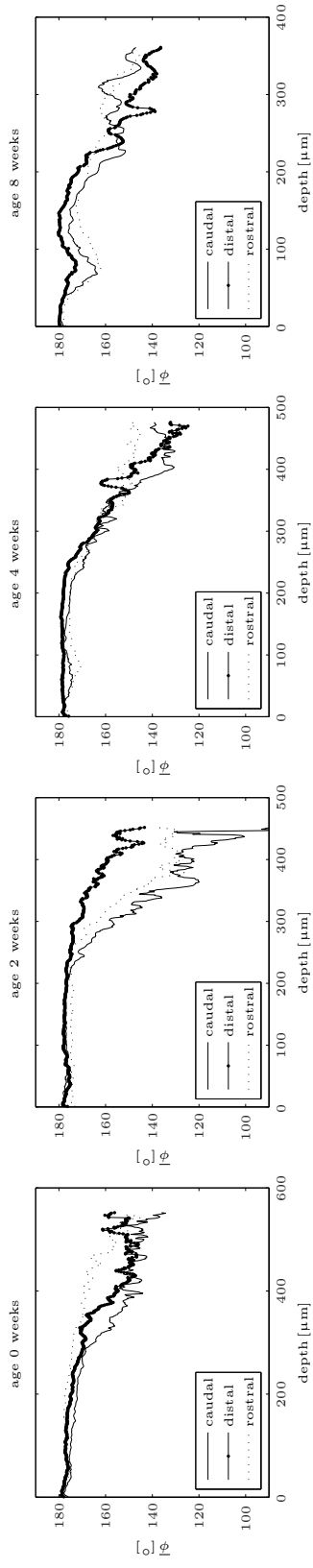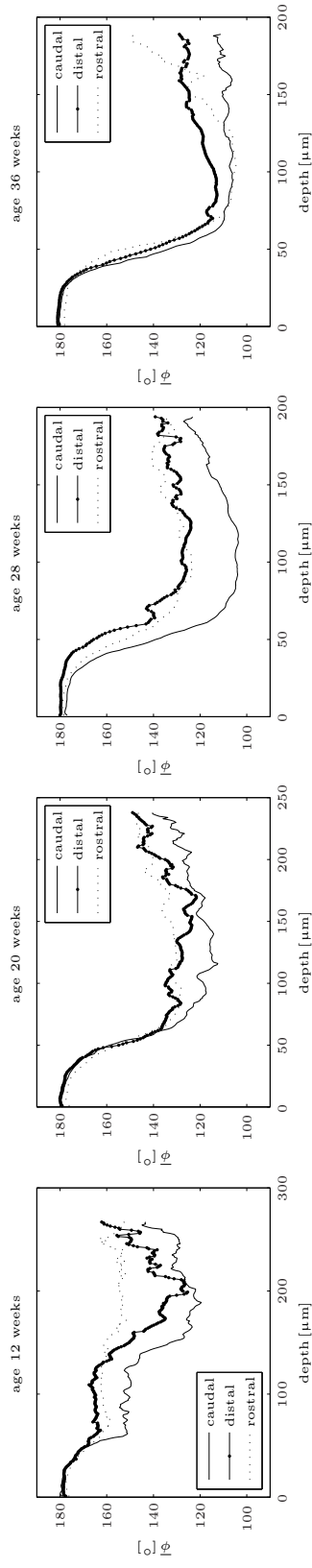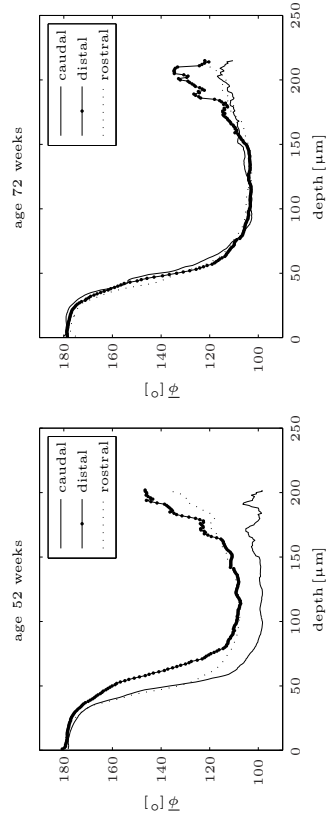

Supplement: Additional file 2 — Orientation patterns for caudal/distal/rostral sites. Average orientation patterns for all ten ages divided in caudal, distal and rostral samples. [file 1471-213X-10-62-S2.PDF]

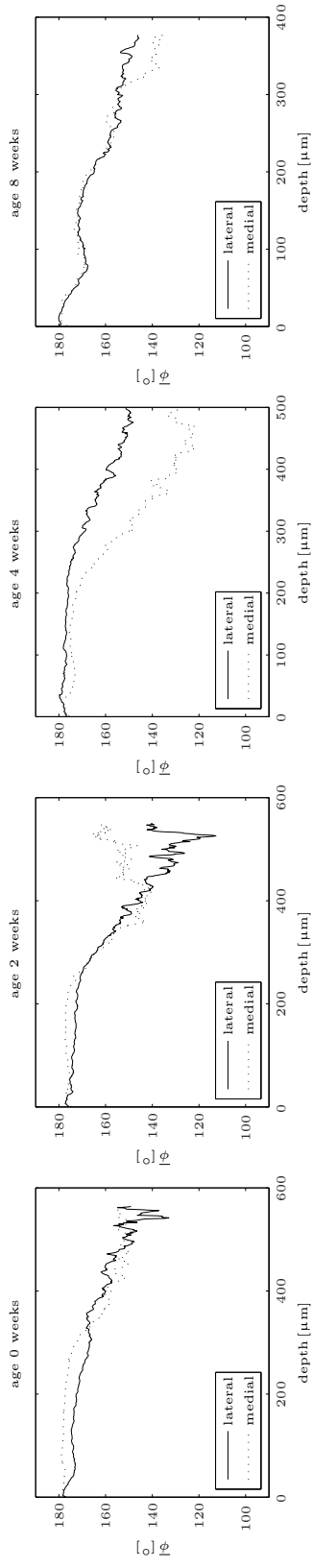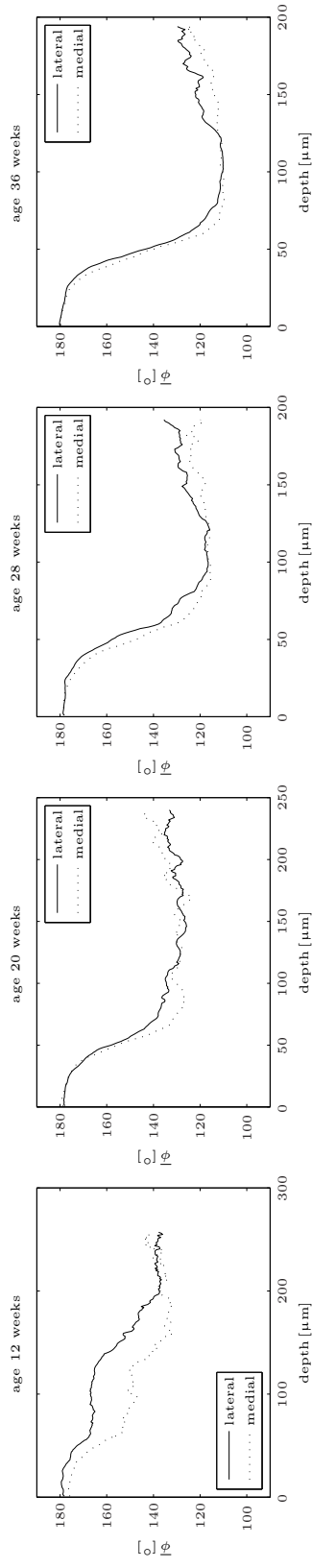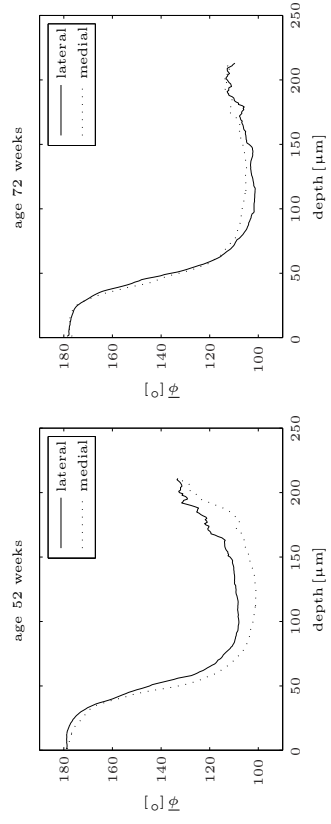

Supplement: Additional file 3 — Orientation patterns for lateral/medial sites. Average orientation patterns for all ten ages divided in lateral and medial samples. [file 1471-213X-10-62-S3.PDF]

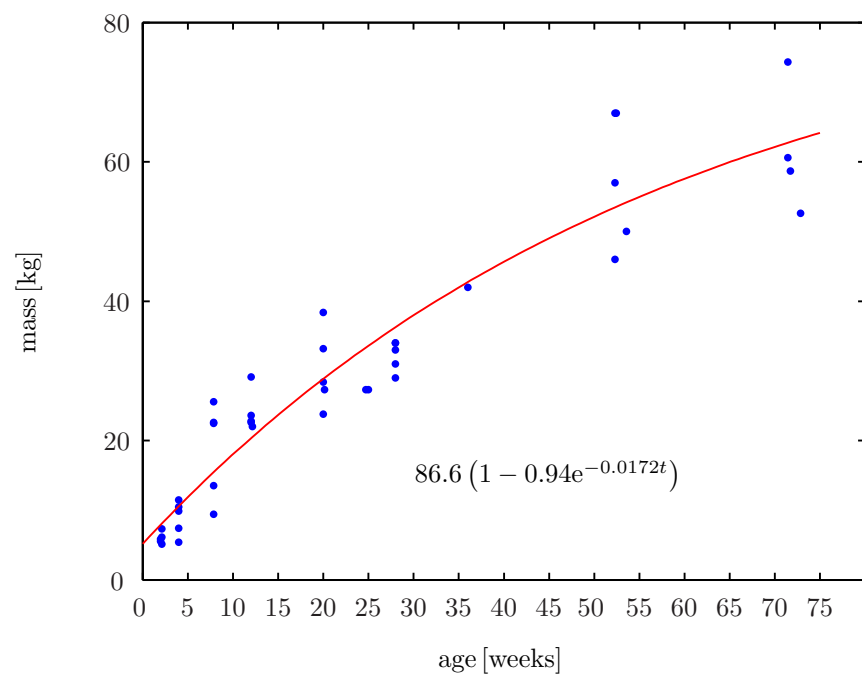

Supplement: Additional file 4 — Slaughter mass. Slaughter mass together with the exponential fit. The mass at 36 weeks is an estimate from the butcher, because these animals were not weighed prior to sacrifice. [file 1471-213X-10-62-S4.PDF]

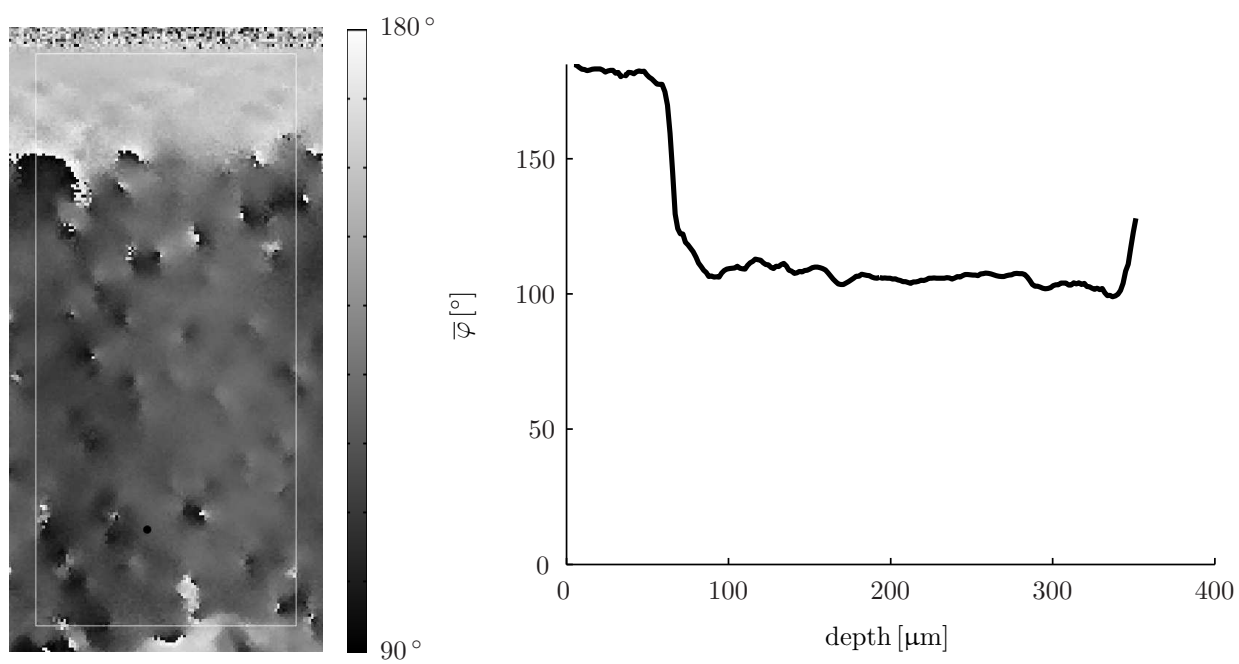

Supplement: Additional file 5 — Constant azimuth in the deep zone. Illustration of the constant azimuth in the deep zone for a 72 week old animal. Left: azimuth PLM image with ROI. The articular surface is on top of the image. Right: corresponding orientation pattern. The azimuth is fairly constant in the deep zone up until the last 3% of the total depth. [file 1471-213X-10-62-S5.PDF]

2 weeks, left hind leg, lateral

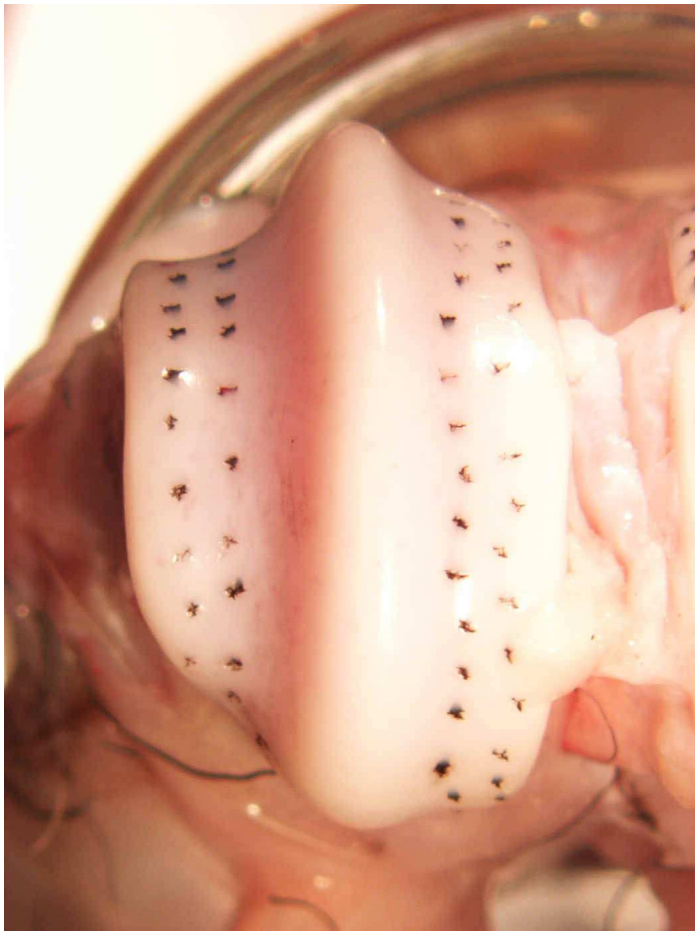

72 weeks, left hind leg, medial

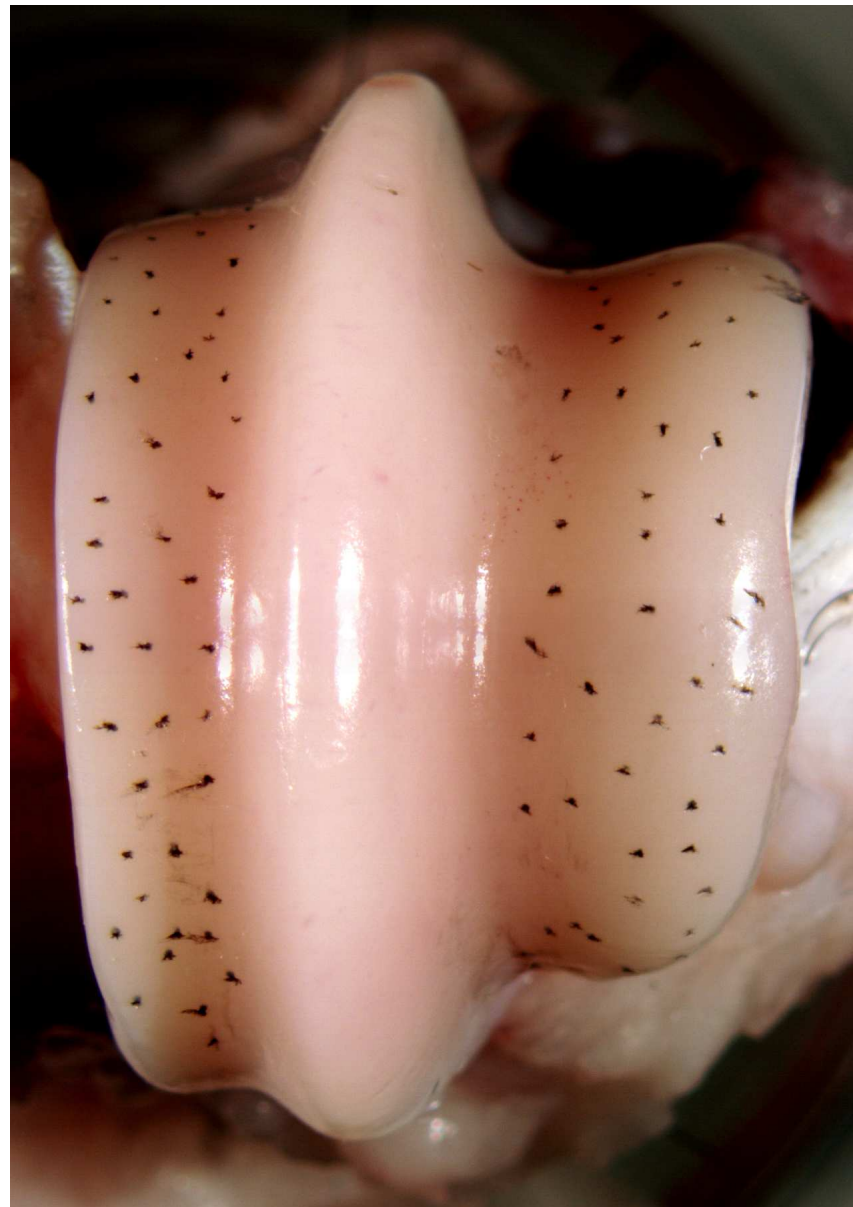

Supplement: Additional file 6 — Illustration of split lines. Left: lateral side of a left hind leg in a 2 week old lamb. Right: medial side of a left hind leg in a 72 week old lamb. [file 1471-213X-10-62-S6.PDF]
